# Emerging Oral Nicotine Products and Periodontal Diseases

**DOI:** 10.1155/2023/9437475

**Published:** 2023-02-10

**Authors:** Dongxia Ye, Irfan Rahman

**Affiliations:** ^1^Eastman Institute for Oral Health, University of Rochester Medical Center, Rochester, NY, USA; ^2^Department of Environmental Medicine, University of Rochester Medical Center, Rochester, NY, USA

## Abstract

Oral nicotine pouches are emerging as a new “modern oral” nicotine product. These prefilled pouches contain nicotine, flavorings, and filling agents that dissolve in the mouth. Nicotine can be derived from tobacco leaf or chemical synthesis. Traces of TSNAs and toxic chromium were detected in the pouch products. This raises the concern about general and periodontal health. This review aims to update the current oral nicotine products research relating to periodontal disease and its relevance in periodontal inflammation. Nicotine interacts with host cells and affects inflammatory responses to microbial challenges. It may directly or indirectly deteriorate periodontal tissues by activating nicotinic acetylcholine receptors, repressing PDL fibroblasts cells, increasing cellular ROS and cytokines/chemokines, growth factors, breaking microbiota balance, and dysregulating miRNAs expression. Studies show that appealing flavorings contained in nicotine pouches pose harm to periodontal innate immune responses and increase penetration of nitrosamines. In addition, flavored ONPs increase the risk of dual or poly-tobacco products among young adults, stacking up detrimental effects on the periodontium. Given the recent growth of users, further studies are needed to elucidate the impact of ONPs, even poly-tobacco use, on systemic and periodontal health. Moreover, policymakers should ensure to avoid generating a new wave of nicotine addiction among youths in the U.S.

## 1. Background

There are 1.3 billion tobacco users globally, and 8 million people die yearly from tobacco smoking [1]. In addition, cigarette smoking contributes to systemic diseases, including cancer, cardiovascular disease, COPD [2], and periodontitis [3]. However, cigarettes are no longer the most popular form of tobacco use, especially among youth smokers, in the United States and globally [4]. In the recent decade, increasing diversity of nicotine-containing products has emerged, including electronic cigarettes (E-cigs) [4], tobacco heating products (THPs) [5], and more recently, oral nicotine pouches (ONPs) [6].

ONPs are preportioned pouches similar to Snus, with a difference from Snus in that there is no leaf tobacco in them [7]. They can be used almost anywhere since they are a convenient, tasty, and discreet way to use nicotine. Large tobacco companies market ONPs as “tobacco-free” [6, 8], “tobacco leaf-free” [8, 9], or “all white” [10]. Along with nicotine lozenges and gum, nicotine pouches are part of the new “modern oral” nicotine product category [11].

ONPs entered the U.S. market in 2016 [10]. As an emerging new product, sales of ONPs rose dramatically from 0.16 million units ($0.7 million) in 2016 to 46 million units ($200 million) during the first six months of 2020 [10]. Because of Nicotine and flavors [8], ONPs appeal to youth and young-adult nonsmokers. As a result, concerns about potential initiation and use among vulnerable young populations are raised. Controversially, nicotine pouches are considered a lower-risk product since they are not combusted and contain no tobacco leaf. However, there is limited evidence to justify its safety. Hence, in this review, we will provide an overview of the current knowledge of oral nicotine products, as it relates to impacts on periodontal health, and identify the potential research gaps.

## 2. Major Constituents and Categories of ONPs

ONPs are composed of a permeable pouch and a nontobacco substrate to which nicotine and flavors are added [6, 7]. The outer pouch material comprises viscose fibers bound together by chemical, heat, or solvent treatment. The nontobacco matrix, constituting approximately 80–90% of the ONPs, mainly comprises moisture content and microcrystalline cellulose [6]. Other food-grade ingredients include nicotine, flavorings, pH buffer, salt, and filling agents [6].

Nicotine pouches are sold in various flavors, e.g., fruit, mint, dessert, tobacco, and so on [8]. Sadiya et al. framed a wheel diagram to show the flavors and flavor categories of ONPs [12]. Fruity and mint/menthol are two of the most widely sold flavors [12]. Flavors and appealing tastes play a significant role in drawing youth to tobacco products and possibly be the primary reason to use the products [13].

The type of nicotine in ONPs is either tobacco-derived or synthetic. Tobacco-derived nicotine (TDN) is extracted from tobacco leaf containing various alkaloids, yielding predominantly (99.3%) (S)-nicotine, whereas synthetic nicotine (SyN) is synthesized chemically, creating racemic (50/50 (R)/(S)) to 99% (S)-nicotine [14].

Federal regulations on ONPs have been through stages. In 2016, the “deeming rule,” namely, “Deeming Tobacco Products To Be Subject to the Federal Food, Drug, and Cosmetic Act,” authorized FDA to regulate all tobacco products that contain TDN, including ONPs [12, 15]. However, to avoid regulation, some companies switched to SyN to keep their products on the market. In 2022, the Consolidated Appropriations Act gave the FDA the authority to regulate the ONPs utilizing SyN [16]. Namely, manufacturers, distributors, importers, and retailers of tobacco products containing TDN or SyN must ensure compliance with applicable requirements. Therefore, any ONP containing SyN or TDN, which has not submitted a premarket application (PMTA) to the FDA, will be removed from the U.S. market [15, 16].

Total nicotine in ONPs exists in ionic forms of both protonated and unprotonated. Protonated nicotine predominates at a pH lower than pH 6.0. Unprotonated nicotine (free or free-base) increasingly predominates as pH levels increase above 6 [17]. Compared with protonated nicotine, nicotine in its unprotonated form passes through oral mucosa epithelium much more rapidly and results in more extensive and rapid increases in blood nicotine levels [18]. Therefore, some ONPs with high unprotonated nicotine may result in greater nicotine dependency.

Most ONPs are labeled with varying nicotine content levels, in either milligram nicotine per pouch or gram. It ranges from 0 (free) to 50 mg total nicotine/pouch [8, 17, 19]. Mallock et al. analyzed 44 nicotine pouches and two nicotine-free pouches products from 20 manufacturers. They found that nicotine contents of products range from not detected nicotine-free to 47.5 mg/pouch [19]. A high level of nicotine is comparable to traditional tobacco products. Therefore, users can acquire adequate nicotine from the pouches to satisfy cigarette cravings.

## 3. Minor Constituents in ONPs

Smokeless tobacco products, including ONPs, usually do not contain typical tobacco-specific nitrosamines (TSNAs). However, toxicological concerns are already present in unsmoked tobacco [20]. TSNAs are present in forms of N-nitrosonornicotine (NNN), N-nitrosoanatabine (NAT), N-nitrosoanabasine (NAB), and 4-(methyl nitrosamino)-1-(3-pyridyl)-1-butanone (NNK) [21]. Mallock et al. detected the existence of TSNAs in 26 of 44 nicotine pouch products. The highest measured concentrations of NNN and NNK were 13 ng and 5.4 ng/pouch [19]. In addition to nicotine and TSNAs, toxic chromium and formaldehyde were detected in some nicotine pouch products [6]. Any trace of toxic elements should not be present because of potential health risks. Exposure to NNN is reported to be associated with promoting esophageal tumors [20]. This raises a special concern for ONPs used in the oral cavity.

In summary, oral nicotine products mainly contains nicotine, flavorings, pH buffer, filling agents, as well as a trace of toxic TSNAs, metal, and formaldehyde. This mix may pose the risk to periodontal health through a different mechanism.

## 4. ONPs Usage and Nicotine Absorption

Nicotine pouches are used by inserting between the lip and gum to release nicotine for 30–60 min per use [11, 19]. Nicotine is absorbed into the blood flow via the oral mucosa, unlike cigarette smoking, heated tobacco, and E-cig, in which nicotine uptake is predominantly via inhalation. The study confirms pharmacokinetics profile of ONPs is mainly oral mucosal absorption and not via swallowing/G.I. absorption [7]. The time of maximum nicotine concentration observed (*T*_max_) for the pouches products ranges from 22–26 minutes to 60 minutes [7, 22]. An *in vivo* study shows that two higher doses of a non-tobacco-based nicotine pouch (ZYN 6 and 8 mg) can deliver nicotine as quickly and to a similar extent as general snus (8 mg) and longhorn moist snuff (18 mg) [22].

## 5. ONPs and Periodontal Health: Inflammation, Dysbiosis, Bone Loss, and miRNAs

Periodontal disease comprises gingivitis, periodontitis, and even implantitis. It is the leading cause of tooth loss and contributes to some systemic diseases [23]. Biologically, periodontal disease results from an imbalance between bacterial virulence and the host defense system. The epidemiological study recognized smoking as a significant risk factor for periodontal disease [24], affecting the prevalence, severity, progression, and clinical treatment response, as well as the long-term success of the implant [25]. Given the well-established effect of tobacco smoke on the periodontium and oral mucosa, it is essential to understand the effects of oral nicotine pouches products. As an emerging product, particular concerns of ONPs have been raised about potential harms to periodontal health that require more investigation.

ONPs users placed the pouches between the lip and gum, similar to chewing tobacco, smokeless tobacco, and snus. Holding the pouches close to the gingival tissue can cause mechanical injury and irritation [26]. Severe attachment loss and gum recession was found among tobacco chewers. References [27, 28] suggest a positive association between smokeless tobacco and clinical severe periodontal disease [29]. In addition, an *in vitro* study shows ONPs extracts cause a toxic response in gingival epithelial cells directly [30]. Thus, physical proximity facilitates the release of the toxic chemical into the local microenvironment, resulting in periodontal problems.

The most plausible hypothesis that explains the relationship between ONPs and periodontal disease is that the primary toxic component of nicotine pouches, namely, nicotine, interacts with host cells and affects inflammatory responses to microbial challenges. It may directly or indirectly deteriorate periodontal tissue. *In vitro* studies show that nicotine exposure significantly activated nicotinic acetylcholine receptor (nAChR) expression [31], repressed periodontal ligament (PDL) fibroblasts cells, and stem cell viability, and increased the generation of cellular reactive oxygen species (ROS) [32, 33]. Increased ROS level subsequently leads to sequential activation of signals, e.g., ERK, JNK, and caspase-3, 9, followed by DNA fragmentation and cell death [32].

Evidence regarding associations between tobacco smoking and the oral microbiome is evolving. A study on oral wash samples from 1204 US adults identified depletion of *Proteobacteria* and enrichment for *Streptococcus* and *Actinobacteria* in smokers [34]. Smoking facilitates the early acquisition and colonization of periodontal pathogens, in the healthy periodontium [35]. In the stage of periodontitis, the subgingival microflora in smokers shifts to a pathogen-enriched community, e.g., *Fusobacterium* [36], *Parvimonas*, *Treponema*, and *Filifactor* [37]. An animal study shows exposure to 250 mg of smokeless tobacco products increased bacterial diversity and abundance of periodontitis pathogenic *Actinomyces*, *Aggregatibacter*, *Streptococcus*, and *Staphylococcus* [38]. As ONP users are most likely former tobacco smokers or dual users, researches are necessary to provide evidence that the use of ONPs may influence the profile of the oral microbiome independently or concomitantly.

Specific bacterial species initiate periodontal disease, then a subsequent local host response to these bacteria is mediated by cytokines and regulated by the recruitment of leukocytes. Nicotine, as the main ingredient in ONPs, plays a vital role in periodontal disease through its influence on cytokine levels. Nicotine enhanced the production of IL-1*β* and IL-8 [39], IL-6, IL-10, and IFN-*γ* [40], PGE2 [41], and decreased MMP2 [42]. The cell coculture model hypothesized that nicotine deteriorates periodontitis by PDL cells deriving CXCL12, recruiting CD4^+^ T cells, then increasing MMP-1, MMP-3, IL-1, IL-6, IL-17, and IL-21 [43].

Alveolar bone loss is a characteristic phenotype of periodontal disease. Smokers with periodontitis have increased probing depth, more significant attachment loss, and even more tooth loss than patients with periodontitis and no smoking history [29]. Smoking adversely affects the alveolar bone height and density in young adults with low tobacco consumption [44]. Nicotine, a significant ingredient of ONPs, is crucial in augmenting bone loss. In periodontally healthy or periodontitis animal models, nicotine causes significant bone loss, more severe in the furcation region [45], and attenuated alveolar bone regeneration by the increased number of osteoclasts in periodontal tissues and upregulated expression of NF-*κ*B ligand [46]. Locally absorbed nicotine recruits inflammatory cells into periodontium, favoring cell-cell interactions [47] and inducing the expression of osteoclastogenesis factors, e.g., RANKL, RANK, TNF-*α*, and IL-1*β*, further the ultimate formation of osteoclasts and more bone degeneration [48].

MicroRNAs (miRNAs), noncoding single-stranded RNAs (∼22 nucleotides), can regulate gene expression and biological function, and play critical roles in the biological process of periodontitis [49]. More specifically, miRNA146a was found to be significantly upregulated in inflamed gingival tissues and suppresses toll-like receptor-mediated nuclear factor-kappaB signaling pathway [50]. Isola et al. demonstrated different gingival crevicular fluid (GCF) miRNA profile in periodontitis and cardiovascular disease (CVD) patients and highlighted GCF miRNA-21 may serve as a potential biomarker for both periodontitis and CVD [51]. Smoking, as a risk factor for periodontal disease, may influence the disease causation and progression through dysregulating miRNAs expression. In vitro study shows nicotine-treated human periodontal ligament cells (PDLCs) presented miRNAs upregulations and downregulations [52]. Those miRNAs involve in the dysfunction of biological processes, molecular function, cellular components, and signaling pathway e.g., NF-*κ*B, epithelial-mesenchymal signaling, and SMAD signaling [52, 53], suggesting ONPs, nicotine-containing products, has the risk of initiating or deteriorating periodontal disease through miRNAs mechanism leading to oral submucous fibrosis. Furthermore, it is needed to investigate the effectiveness of tacrolimus to reverse the immunoinflammatory condition due to its inhibition of the transcription and production of proinflammatory factors [54].

## 6. Risk of Constituents Other than Nicotine to Periodontal Health

Additionally, constituents except for nicotine in ONPs drive concern about general and oral health, such as flavorings, TSNAs, and so on. The US FDA Tobacco Control Act of 2009 banned all characterizing cigarette flavors except menthol. ONPs are not in the range of this Act. Nevertheless, there are more than 15,000 flavor options [55] available for smokeless products, e.g., 2,3-hexanedione (creamy; fruity), ethyl butyrate (fruity; apple), DL-menthol (menthol; minty). [56]. Those flavorings induce adolescent smokers to develop physical nicotine dependence quickly and increase emotional attachments to cigarettes [57] by increasing positive and decreasing negative subjective experiences [58]. In addition, menthol additives impact human PDL fibroblasts' vitality and proliferation [59, 60] through increased oxidative stress, and proinflammatory and prosenescence responses [59]. It suggests flavor additives in ONPs pose the risk to periodontal health.

Flavored nicotine products could induce microbial dysbiosis in the oral cavity and periodontium, inhibit local innate immune responses, and cause the pathogenesis of periodontitis resulting from the interplay between respiratory microbiota and innate immunity [61]. Furthermore, the flavor of menthol facilitates penetration of toxic chemicals, e.g., nitrosonornicotine (NNN) and nicotine, across buccal and floor-of-mouth mucosa [62], posing the risk to oral soft tissue lesion.

The study found increased risk of dual and poly tobacco product use is associated with flavored tobacco [63]. This implies a new public health risk because detrimental effects will be stacked up with dual or ploy tobacco product use. It is evidenced in a pilot study that dual smokers presented more inflammatory factors in saliva and gingival crevicular fluid compared to nonsmokers [64]. The impact of tobacco poly-use on periodontal health still needs more investigation.

## 7. Conclusion

**Figure 1:**
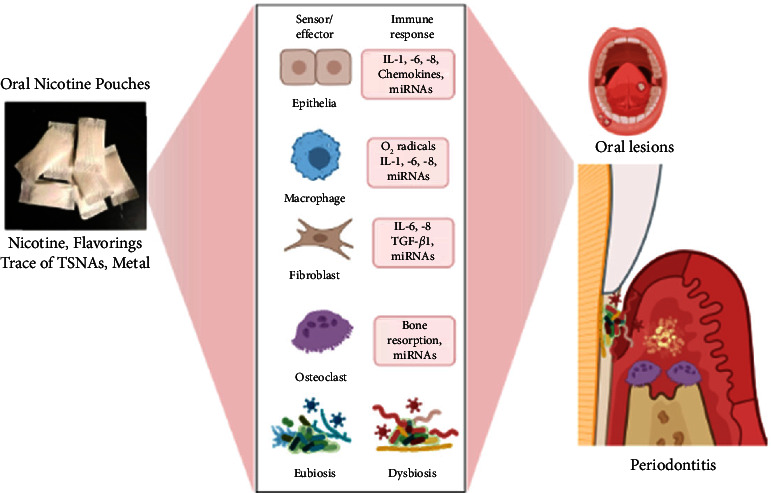
Schematic mechanism showing ONPs cause oxidative stress/inflammation/immune dysfunction, dysbiosis, and miRNAs dysregulation on periodontal health, which was prepared using biorender.

In summary, emerging oral nicotine pouches pose significant health risks (Figure 1), especially for adolescents and young adults. Therefore, further studies are warranted to assess how oral nicotine pouch and polyproducts use affect systemic and periodontal health, and toxicity, identify new biomarkers, e.g., miRNAs and inflammatory mediators, profibrotic mediators, for early diagnosis, and explore new anti-inflammatory agents, e.g., tacrolimus, for treating periodontal and oral diseases. In addition, regulatory policymakers should ensure to avoid generating a new wave of nicotine addiction among youths in the U.S.

## Data Availability

No underlying data were collected or produced in this study.
